# Functional characterization of HIV-1 Nef proteins with heterologous membrane targeting signals

**DOI:** 10.1186/1742-4690-10-S1-P36

**Published:** 2013-09-19

**Authors:** Miriam M Geist, Xiaoyu Pan, Oliver T Fackler

**Affiliations:** 1 Department of Infectious Diseases, Virology, University Hospital Heidelberg, Heidelberg, Germany

## Background

The Nef protein is a pathogenicity factor of HIV-1 that substantially increases virus replication *in vivo* by modulation of multiple host cell transport and signal transduction processes. Nef resides at the inner leaflet of the plasma membrane (PM), on membranes of endocytic and biosynthetic transport vesicles as well as in the cytoplasm as non-membrane associated form. Biological activities of Nef are generally thought to depend on its membrane association, which is mediated by the N-terminal SH4 (Src homology) domain, consisting of myristoylation and a stretch of basic residues. However, how targeting of Nef to select subcellular host cell membranes is achieved and which subcellular pools of Nef are involved in individual activities of the viral protein is not clear.

## Materials and methods

A series of chimeric Nef proteins was constructed in which Nef’s SH4 domain was replaced by heterologous SH4 domains of Src family kinases, or different organelle targeting sequences, leading to proteins with distinct subcellular localization, biosynthetic transport pathways and membrane affinities. These Nef variants were characterized for their subcellular localization and membrane association, as well as tested for downregulation of cell surface receptor molecules (CD4 and MHC-I), inhibition of actin rearrangements, and retargeting of Lck.

## Results

The Nef constructs containing the different SH4 domains displayed specific and distinct patterns regarding their subcellular distribution, transport pathways to the PM, and extend of membrane association. Surprisingly, despite those remarkable differences, the different Nef variants were all functional in the biological assays conducted. In contrast, organelle targeted Nef variants, which were membrane-associated with Nef being exposed to the cytoplasm but localized to unphysiological subcellular sites such as mitochondria or peroxisomes, proved completely inactive.

## Conclusions

The full functionality of all SH4 domain containing constructs indicates that membrane association *per se* but not the specific steady state subcellular localization, membrane affinity, or anterograde transport pathway of Nef is essential for its biological activity. This would be consistent with a scenario in which membrane-bound Nef acts as a “titrating factor” that recruits interacting proteins to its site of localization to disrupt host cell homeostasis. However, this hypothesis is excluded by the lack of function of the organelle targeted Nef chimera. Together this suggests that undergoing the full membrane-associated “life cycle” from biosynthesis, PM delivery and subsequent internalization might be required for Nef to exert its biological activities. To test this, we are currently establishing an experimental system in which Nef transport can by synchronized and initiated from selected subcellular sites.

